# Hemophilia carrier’s awareness, diagnosis, and management in emerging countries: a cross-sectional study in Côte d’Ivoire (Ivory Coast)

**DOI:** 10.1186/s13023-019-1005-9

**Published:** 2019-02-01

**Authors:** Catherine Lambert, N’ Dogomo Meité, Ibrahima Sanogo, Sébastien Lobet, Eusèbe Adjambri, Stéphane Eeckhoudt, Cedric Hermans

**Affiliations:** 1 Hemostasis and Thrombosis Unit, Division of Hematology, Cliniques universitaires Saint-Luc, Brussels, Belgium; 2 Division of Clinical Hematology, Centre Hospitalier universitaire de Yopougon, Abidjan, Côte d’Ivoire; 3 Division of Physical Medicine and Rehabilitation, Cliniques universitaires Saint-Luc, Brussels, Belgium; 4 Université catholique de Louvain, Secteur des Sciences de la Santé, Institut de Recherche Expérimentale et Clinique, Neuromusculoskeletal Lab, Brussels, Belgium; 5 Hematology unit of the Central Laboratory, Centre Hospitalier universitaire de Yopougon, Abidjan, Côte d’Ivoire; 6 Hemostasis and Thrombosis Laboratory, Division of Biological Chemistry, Cliniques universitaires Saint-Luc, Brussels, Belgium

**Keywords:** Carrier of hemophilia, Ivory Coast, Developing countries, Data collection, WFH twinning program

## Abstract

**Background:**

Little data is available on awareness of hemophilia carrier condition or associated bleeding risk and management in Sub-Saharan African countries. This study sought to identify hemophilia carriers in Côte d’Ivoire in order to collect data on demographics, bleeding phenotype, and laboratory results. Another purpose was to provide Ivorian hemophilia carriers with counseling on their risk of bleeding and of having children with hemophilia. A 12-month prospective study was conducted involving Ivorian hemophilia carriers recruited trough pedigree analysis pertaining to 81 hemophilia patients followed-up at the Yopougon Hemophilia Treatment Center in Abidjan. They were assessed using in-depth interviews, pedigree analysis, and laboratory testing.

**Results:**

Sixty-one subjects comprising 27 obligate and 34 possible carriers were recruited. None had previously been assessed, with 64% unaware of their carrier status despite a familial history of hemophilia in 69%. The most frequently reported bleeding symptom was menorrhagia (31%). Prolonged bleeding was reported after vaginal delivery in 19.6%, post-surgery in 4.9%, and post-dental extraction in 4.9%. Only one carrier was treated with tranexamic acid, with no other hemostatic therapy recorded. The median (range) clotting FVIII was 0.85 IU/mL (0.24–1.90 IU/mL) and FIX 0.60 IU/mL (0.42–1.76 IU/mL) in hemophilia A and B carriers, respectively. HA carriers had a FVIII < 0.5 IU/mL in 12.5%.

**Conclusions:**

This study highlights the need of implementing care for hemophilia carriers in developing countries, and the high value of pedigree analysis for carrier identification, along with the relevance of diagnosis, treatment, and education of carriers, families, and caregivers.

## Introduction

Hemophilia is a congenital X-linked recessive bleeding disorder causing low levels of Factor VIII (FVIII, hemophilia A [HA]) or Factor IX (FIX, hemophilia B [HB]), occurring in approximately 1 in 5000 for HA and 1 in 20,000–30,000 for HB) live male births [1], with a similar incidence across ethnic populations [2]. The condition affects men while their female relatives are likely to be hemophilia carriers. It has been estimated that for each male with hemophilia, there are five potential female carriers [3]. Carriers are at risk of having sons with hemophilia but may also carry a bleeding risk [4].

A wide range in clotting FVIII or FIX levels is observed in carriers, independently of hemophilia severity within the family [5], which is attributed to the lyonization phenomenon (random X-chromosome inactivation during embryonic life) [6]. In HA carriers, Factor VIII levels display a considerable variability, ranging from very low to the upper limit of normal [7]. Carriers in the mild hemophilia range are defined by factor levels < 30 IU/mL, though there is no consensus about this threshold, and are labeled “symptomatic carriers” [8]. Carriers may show an increased bleeding tendency [5], even those with normal FVIII levels [9, 10]. Assessing bleeding risk is therefore mandatory in all obligate or potential carriers [5].

Previously, pedigree analysis and clotting FVIII/IX levels were applied for diagnosing hemophilia carriership [11]. Since the 1980’s, DNA analysis has been available, which ascertains hemophilia carriership in most developing countries [12, 13]. Currently, comprehensive care in hemophilia patients comprises carrier factor testing, genetic counseling, and prenatal diagnosis [5, 8, 14]. Detection of carriers is likewise relevant considering the 3.5–4% incidence of intracranial bleeding in affected male newborns [14].

In developing countries, carrier detection proves even more crucial for early hemophilia diagnosis in children, because they often die early in life due to the lack of diagnostic opportunities, absence of care facilities, and limited access to clotting factor concentrates (CFC) [15]. As circumcision generally represents the first surgical intervention in African boys with hemophilia, it is a major cause of bleeding and death [16]. Unawareness of carrier condition and bleeding risk, as well as healthcare givers’ ignorance concerning appropriate management modalities, are also major issues of concern in these countries [4, 17]. In Sub-Saharan Africa, little is known about hemophilia carriers [3, 4, 18, 19], with only very limited access to molecular testing [18, 20], and pedigree and clotting FVIII and IX analyses being often the principal screening and assessment tools.

In 2015, a twinning partnership was established between the Ivorian hemophilia treatment center (HTC) of the *Centre Hospitalier universitaire (*CHU) of Yopougon in Abidjan and the international HTC of the *Cliniques universitaires Saint-Luc* in Brussels, Belgium. This collaboration has provided the opportunity to identify and assess potential and obligate hemophilia carriers among families of patients with hemophilia (PWH) in this location.

## Study objectives

This study aimed to identify obligate and potential carriers of hemophilia in Côte d’Ivoire and collect detailed information on their demographics, pedigree, bleeding phenotypes, and laboratory results. Yet, its ultimate goal was to provide Ivorian hemophilia carriers with appropriate counseling on the risk of bleeding and of having children with hemophilia.

## Patients and methods

### Patients

Between January and December 2017, we invited all the ≥12-year-old female Ivorians, identified as HA or HB carriers, to participate in this study conducted at the Yopougon HTC. Candidates were recruited via detailed family trees of 81 PWHs followed-up at the HTC. The carriers, their parents, or legal guardians provided written informed consent for participating in the study.

### Methods

This was a single-center cross-sectional study conducted from January to December 2017 on the Ivorian hemophilia carrier population. Data were prospectively collected during standardized consultations held by Belgian and Ivorian teams including a hematologist and hemostasis laboratory member.

For each carrier, an in-depth face-to-face interview was performed gathering data on carrier status awareness, socio-demographics, educational activity, employment, medical, obstetrical, and surgical history, bleeding symptoms and circumstances, requirement and indication for whole blood transfusion, as well as co-medications including estroprogestative contraception, iron supplementation, and hemostatic and antifibrinolytic agents’ usage. A specific attention was paid to menorrhagia assessed by the bleeding questionnaire described by *Tosetto* et al, 2006. Women who had one point or more for this item were considered as having menorrhagia.

To identify carriers in each family, a detailed pedigree was drawn. Efforts were made to investigate as many generations as possible. Of note is that the family trees were repeated at each evaluation of several different members of the same family, whether carriers or PWHs. This allowed us to establish exhaustive and validated trees, after merging, correcting and updating steps. Mendelian laws were applied to determine the probability of being a carrier. The carriers were categorized as obligate or possible/probable carrier, based on the pedigree, the level of FVIII or FIX and according to the WFH definitions [21].

The biological workup comprised a complete blood-count (Cell-Dyn Ruby, Abbott) and FVIII and FIX activity measurements by one-stage assay method on a semi-automated coagulometer (Option 4 Plus, Biomerieux) using human plasma immunodepleted for FVIII and FIX (HemosIL, Werfen).

### Statistical analysis

All data were entered into a structured electronic medical record and analyzed using SigmaStat and SigmaPlot (Systat Software Inc., USA). The pedigrees were drawn using the Progeny Free Online Pedigree Tool from Progeny Software (Progeny Software LLC, FL., USA).

## Results

Based on pedigree analysis of 81 Ivorian PWHs, we identified 83 obligate carriers (12 for HB and 71 for HA) and 248 possible carriers (30 for HB and 218 for HA), resulting in an average of four carriers per PHW. A total of 61 carriers for severe and moderate hemophilia A and B were included with 27 obligate carriers (3 for HB and 24 for HA) and 34 possible carriers (2 for HB and 32 for HA) (Table 1). The median (range) age at evaluation was 34 (13–58) years. None had previously been assessed nor were clotting FVIII or IX levels measured prior to the WFH’s twinning program, with 64% unaware of their carrier status.

**Table 1:** Distribution of hemophilia carriers in Ivory Coast

Obligate carriers	n =	Possible carriers	n =
Obligate carriers for severe HA	23	Possible carriers for severe HA	29
Obligate genetic carriers for moderate HA	1	Possible carriers for moderate HA	3
Obligate genetic carriers for severe HB	3	Possible carriers for severe HB	2
Total	27		34

Obligate and possible carriers for hemophilia were determined by pedigree analysis

### Socio-demographics and medical history

Regarding ethnicity, carriers were classified as Akan (36%), Krou (36%), Mandé (14,8%), Gour (10%), or other (3,2%). Most were living in or around Abidjan (54%), had a professional activity (47.6%), were homemakers (36%), with the remainder either students or in professional training (16.4%). Their marital status was as follows: married (44%), single (28%), in couple (15%), divorced (11%), or widowed (2%). Malaria, which is endemic in Ivory Coast, was the most common medical condition [22]. The HIV prevalence in carriers (3,3%) was similar to that of the Ivorian general population [23]. Surgical histories were recorded in 19,7%, with cesarean and appendectomy as the most common procedures (Table 2).

**Table 2:** Medical and surgical history

Medical event	*n* = 26	Surgical procedure	*n* = 14
Malaria	6	Caesarian section	7
Peptic ulcer/gastritis	5	Appendicectomy	6
Hypertension	4	Inguinal hernia repair	1
HIV infection (on retroviral therapy)	2		
Ectopic pregnancy	2	Tooth extraction (non-surgical)	*n =* 15
Uterine fibroid	2		
Asthma	1		
Allergies	1		
Arthrosis	1		
Glaucoma	1		
Bowel disease	1		

### Pedigrees

Detailed pedigrees were obtained through 142 interviews with 61 carriers and 81 PWHs. The 61 carriers studied were issued from 32 families. A familial form of hemophilia was present in 20 families involving 42 carriers and a sporadic form, defined as cases with no other PWH – whether alive or deceased - within the family, in 12 families involving 19 carriers. In familial forms, the median number of affected relatives per carrier was 4 (range 2–11). The median (range) number of obligate carriers and possible carriers per family for familial forms was 2 (0–4) and 4 (1–14), respectively. In sporadic forms, the median (range) number of possible carriers per family was 3 (1–6). Among these 20 families, 39 PWH death cases were identified, 12 of whom were related to circumcision and 7 to intracranial hemorrhage. The median number of children per carrier was 2 (range 0–9) and that of sons affected with hemophilia per carrier was 1 (range 0–6).

### Bleeding symptoms and circumstances

The most frequently spontaneous bleeding symptom was excessive or prolonged menstrual bleeding, with 31% prevalence among the whole carrier population and 26% prevalence in the obligate carrier group. Other unprovoked hemorrhagic symptoms, such as nose bleeds, cutaneous bruising, gum bleeding, or hemarthrosis, had < 5% prevalence (Table 3). Prolonged bleeding after vaginal delivery or miscarriage was reported in 19.6 and 2.2% of the 46 carriers, respectively, during previous pregnancies involving 160 deliveries and 6 miscarriages. Postoperative bleeding was recorded in 4.9% of cases upon 2 cesareans and 1 episiotomy (stitches rupture). A prolonged bleeding after tooth extraction was mentioned in 4.9%. Of note, these tooth extractions were simple, non-surgical.

**Table 3:** Spontaneous bleedings in Ivorian hemophilia carriers

Event	All carriers (n= 61)	Obligate carriers (n= 27)	Possible carriers n= 34)
	event/total (%)	event/total (%)	event/total (%)
Heavy/prolonged periods	19 (31%)	7 (26%)	12 (35%)
Nose bleeding	3 (5%)	3 (11%)	2 (6%)
Gum bleeding	3 (5%)	1 (3,7%)	2 (6%)
Bruising	2 (3%)	1 (3,7%)	1 (3%)
Joint bleeds	0 (0%)	0 (0%)	0 (0%)

### Treatments

No carrier had previously been treated with CFCs, cryoprecipitates, or fresh frozen plasma. Whole blood transfusion was reported in 3 carriers (1 symptomatic obligate carrier for HB and 2 obligate carriers for HA), 1 after vaginal delivery, 1 after cesarean, and 1 after a complicated episiotomy. No transfusion was recorded after spontaneous bleeding. One carrier reported a blood transfusion for anemia in childhood. Tranexamic acid was used on-demand by only one symptomatic carrier for severe HA while experiencing menorrhagia, mucocutaneous bleeding, and postpartum hemorrhage. None of the HA carriers had previously been assessed nor treated with Desmopressin (DDAVP), or preventively treated with hemostatic therapies. Iron supplementation was recorded in 3.3%. Eighteen % were on hormonal therapy, using estroprogestative pills (9), implants (1), or subcutaneous injections (1). Other treatments comprised paracetamol in 14.8%, anti-malarial drugs in 4.9%, and antiretroviral therapy in 3.3%. No one reported using anti-inflammatory drugs.

### Laboratory results

In 38% of participants, clotting factor activity had previously been determined in the 2016 WFH’s twinning program, with the lowest value considered for this study. The median (range) clotting FVIII level in the 56 HA carriers was 0.85 IU/mL (0.24–1.90 IU/mL). The median (range) clotting FVIII level in obligate and possible HA carriers were 0.75 IU/mL (0.24–1.90 IU/mL) and 0.92 IU/mL (0.60–1.57 IU/mL), respectively. Overall, 7 (12.5%) HA carriers had a FVIII < 0.50 IU/mL. In the 5 HB carriers, the median (range) FIX level was 0.60 IU/mL (0.42–1.76 IU/ml), with 2 (40%) displaying FIX < 0.50 IU/mL. Bleedings in correlation with factor level decreases are listed in Table 4. Table 5 details FVIII and IX levels in carriers, along with the HA carrier percentages on hormonal therapy.

**Table 4:** Clotting FVIII and IX levels among Ivorian hemophilia carriers

Clotting Factor VIII level	More than 0.60 IU/ml (%)		Between 0.41 and 0.60 IU/mL (%)		0.40 IU/ML or below (%)	
		(No on hormonal therapy)		(No on hormonal therapy)		(No on hormonal therapy)
Carriers of HA (n=56)	78.6%	8	12.5%	1	8.9%	0
Obligate (n=24)	30.4%	3	3.6%	0	8.9%	0
Possible (n=32)	48.2%	5	8.9%	1	0%	0
Clotting Factor IX level	More than 0.60 IU/ml (%)		Between 0.41 and 0.60 IU/mL (%)		0.40 IU/ML or below (%)	
Carriers of HB (n=5)	40%		60%		0%	
Obligate (n=2)	0%		60%		0%	
Possible (n=2)	40%		0%		0%

**Table 5:** Spontaneous and post-operative bleedings according to FVIII and FIX levels copie

Bleeding event	Clotting Factor VIII or IX level (IU/mL)		
	More than 0.60 IU/mL (%) (total 46)	Between 0.41 and 0.60 IU/mL (%) (total 10)	0.40 IU/mL or below (%) (total 5)
Heavy/prolonged periods	16 (35%)	1 (10%)	2(40%)
Nose bleeding	2 (4%)	2(20%)	1(20%)
Gum bleeding	2 (4%)	0 (0%)	1(20%)
Bruising	1 (2%)	0 (0%)	1(20%)
Post-partum	6 (13%)	2(20%)	1(20%)
Post-abortum	1 (2%)	0 (0%)	0 (0%)
Post-operative	0 (0%)	2 (20%)	1(20%)

The median (range) hemoglobin level was 11.9 g/dL (8.81–14.8), with a level < 10 g/dL found in 8.8%. The median (range) platelet count was 243 × 10 [9]/L (57–415). Blood counts were missing in 4.

## Discussion

Limited research has been conducted so far in Sub-Saharan countries on hemophilia carriers [3, 18, 19, 4]. Accurate and detailed data in this population are highly relevant to prevent and treat bleedings appropriately, offer genetic counseling, and diagnose hemophilia early in life in boys, with the aim to avoid death and severe complications, mainly due to intracranial bleeding and circumcision. This is the first prospective study focused on detection and assessment of Ivorian hemophilia carriers.

Pedigree analysis rendered it possible to identify 331 possible or obligate carriers, of which 61 were considered for analysis. Considering the theoretical 400 carriers linked to the 81 PHWs from the HTC of Yopougon, pedigree was of great value to target carriers within PWH’s families. In the absence of molecular testing availability, pedigree is a cost-effective and useful tool for assessing the carrier status of females from PWH’s families and can be of a great help in providing genetic counselling [24]. Family trees were sometimes large and complex to build up. It was thus repeated trough several family members, be merged, corrected, and updated in order to obtain quality data.

Despite numerous affected relatives or hemophilia-related deaths (31% after circumcision), at least in the familial forms, 64% of participants were unaware of their carrier status. None had ever been clinically assessed, nor had FVIII or IX level measurements been conducted prior to the twinning program. No carrier had ever had DDAVP testing, with only one carrier previously treated with tranexamic acid for bleeding. These data highlight the lack of the carriers’ knowledge of hemophilia inheritance and their bleeding risks, along with the ignorance of healthcare providers concerning their increased bleeding risk and bleeding management. This highly-relevant unawareness about carrier condition has already been reported in studies conducted in India and South Africa [4, 16, 25].

In our study, 44.3% of carriers reported spontaneous bleeding, with menorrhagia the most common symptom. This is in line with data from other studies focused on hemophilia carriers [9, 10], several of which were conducted in African countries [3, 4]. The rate of other unprovoked bleedings, such as epistaxis 5%, gingivorrhagia 5%, and bruising 3%, proved low in our cohort, in comparison with previous studies [4, 10]. No hemarthrosis was reported. Postpartum bleeding frequency, reported to vary in the scientific literature [4, 10, 19], was estimated at 19.6% in our cohort. Postoperative hemorrhage was reported in 4.9% of cases and dental extraction hemorrhage in 20%. In published reports, there were higher postsurgical bleeding rates, occurring mainly after tonsillectomy [5] and tooth extraction [9, 10]. Several studies conducted in Africa demonstrated lower tooth extraction bleeding rates [4, 19]. In our study, however, the number of surgical procedures was limited, with only appendectomy, caesarian section, and non-surgical tooth extractions considered. Another hypothesis to account for the low frequency of hemorrhagic symptoms could be the fact that it was the first time Ivorian carriers were questioned about their bleeding symptoms, or due to their unawareness of increased bleeding risk.

Although most carriers exhibited normal-range factor levels, a significant proportion displayed decreased factor activity (between 40 and 60 IU/mL in 12.5% ​​of HA carriers and 60% of HB carriers and < 40 IU/mL in 9% of HA carriers and 0% of HB carriers). Bleeding was recorded in the three factor level categories. Our findings are consistent with literature data indicating that carriers may exhibit variable factor levels [7], with an increased bleeding tendency for those with clotting factor levels at the distribution extremes (< 0.40 IU/mL), with mildly-reduced clotting factor levels (0.41–0.60 IU/mL) [5] or even normal FVIII activity [9, 10].

Our study displays several limitations: 1) molecular testing was unavailable to formally establish or confirm the carrier diagnosis; 2) factor levels were measured only once in 62% and twice in 38%, and this measurement could not be repeated at distance of hormonal therapy; 3) the factor activity testing was performed using only a one-stage coagulation assay; 4) we had no information on blood group and von Willebrand antigen/activity; 5) anemia interpretation proves difficult in Ivory Coast where malaria is endemic [22].

Given our study data and within the frame of the WFH’s twinning program, actions have been implemented at Yopougon’s HTC designed to improve the care of hemophilia carriers. All the participants were personally informed, using either oral or written support, about their carrier status, bleeding risk, preventive and therapeutic hemostatic measures to apply if necessary, need for a medical follow-up especially during pregnancy and upon delivery, as well as the relevance to screen all male children for hemophilia early in life and at least before circumcision. The seven carriers with a FVIII level < 50 IU/mL underwent DDAVP testing, and the use of tranexamic acid was encouraged as necessary. Educational booklets and workshops were developed and provided, highlighting the need to actively screen carriers among PWH’s families, and assess their bleeding risk by factor level measurements.

Possible and obligate carriers were provided with the same information, as there is actually no regular access to DNA analysis in Côte d’Ivoire. Mother education proves to be the cornerstone towards improving PWH’s care, as gender plays a key role in shaping the burden of care in Africa, with women more likely than men to take on caregiving activities [3]. Moreover, these educational sessions offered the opportunity to address carriers’ personal and familial psychosocial burden - not to be underestimated in developing countries [4]. This awareness campaign is scheduled to be repeated over time, with its outcome monitored trough surveys and corresponding data published.

## Conclusion

The current study illustrates the lacking awareness of the hemophilia carrier condition and its implications for carriers, PWH’s families, and medical community in Côte d’Ivoire and by extension in other developing countries. The data highlight the need for implementing dedicated care and developing guidelines taking in account local conditions and priorities, enabling carriers to be identified, their factor levels to be assayed, and counseling on the possible chances of bleeding and having children with hemophilia.

This work underlines the significance of repeated and detailed pedigrees in carrier screening, especially in countries with limited molecular testing. Providing access to individual assessment, establishing appropriate hemostatic treatment plans, and using adjunctive hemostatic drugs (DDAVP, antifibrinolytics) should be actively promoted. Education and information of the carriers, their families, and healthcare givers form the basis enabling us to carry out this major project in Côte d’Ivoire.

This research demonstrates the benefits and strengths of a multidisciplinary and comprehensive approach in holistic hemophilia care involving all family members including females in the WFH twinning program setting.

## Abbreviations

BU: Bethesda units
CFC: Clotting factor concentrates
DDAVP: Desmopressin
HA: Hemophilia A
HB: Hemophilia B
HTC: Hemophilia Treatment Center
PWH: patient with hemophilia
